# A Treatise on the Diagnosis and Treatment of Diseases of the Chest. Part. I. Diseases of the Lung and Windpipe

**Published:** 1838-04

**Authors:** 


### Art. VII.

1. *A Treatise on the Diagnosis and Treatment of Diseases of the Chest.* Part I. *Diseases of the Lung and Windpipe.* By William Stokes, m.d. m.r.i.a., Physician to the Meath Hospital, &c.—*Dublin*, 1837. 8vo. pp. 557.

2. *Notes et Additions au Traité de l'Auscultation Médiate de* Laennec. Par M. Meriadec Laennec, d.m.p. &c., et M. Andral, Professeur à la Faculté de Médecine de Paris, Médecin de l'Hôpital de la Charité, &c.
*Notes and Additions to the Treatise on Mediate Auscultation of* Laennec. By M. Meriadec Laennec, m.d. Paris, &c.; and M. Andral, Professor of the Faculty of Medicine of Paris, Physician to the Hospital of La Charité, &c.—8vo. pp. 510; *with two coloured Plates. Paris*, 1836.

3. *Observations on the Surgical Pathology of the Larynx and Trachea, &c.* By Wm. Henry Porter, a.m., Vice-President and Professor of the Theory and Practice of Surgery in the Royal College of Surgeons in Ireland; Surgeon to the Meath Hospital, &c.—*Dublin*, 1837. 8vo. pp. 280.

4. *A Treatise on the Diseases and Injuries of the Larynx and Trachea, &c.* By FREDERICK RYLAND, Surgeon to the Town Infirmary, Birmingham.—*London*, 1837. 8vo. pp. 338; *with Plates.*
5. *Traité pratique de la Phthisie laryngée de la Laryngite chronique, et des Maladies de la Voix.* Par MM. A. TROUSSEAU et H. BELLOC. *Ouvrage couronné par l'Académie Royale de Médecine.*—8vo. pp. 488. *Paris*, 1837.

*Practical Treatise on Laryngeal Phthisis, Chronic Laryngitis, and Affections of the Voice.* By Messrs. A. TROUSSEAU and BELLOC, &c. —*With Engravings.*

IN a former Number, (No. VIII., Art. I.) we were occupied so fully by the valuable work of Dr. Stokes and the interesting Notes of M. Andral, that we had space only to mention the names of the other works which stood at the head of our article. To these, the chief subjects of which are the diseases of the larynx and trachea, we now add another volume, from the other side of the Channel; but, before proceeding to notice them, we must say a few words on the concluding section of Dr. Stokes's work on Diseases of the Pleura, and also on the Notes of M. Andral on Diseases of the Heart.

We believe that Dr. Stokes is the first writer who has recognized a fibrous layer in the pulmonary pleura; although an account of it was published by M. Bazin, (see our last Number, p. 220,) previously to the appearance of Dr. Stokes's treatise. This membrane may be shewn by making a very slight incision, in the shape of the letter U, on the surface of a distended lung, and gently drawing back the serous membrane: the fibrous, which is much stronger, is left smooth and shining under, and may also be separated from the irregular surface of the pulmonary tissue. This fibrous tunic, although increasing the strength of the pleura, does not prevent it from yielding to a certain degree of distension, as is seen in the displacement of the mediastinum by pleuritic effusions and dilatation of the pericardium in the analogous affection of this membrane.

But the most important part of Dr. Stokes's observations is on paralysis of the intercostal muscles and diaphragm, as an effect of the inflammation of the pleura, and as a cause of the yielding of these muscles to the pressure of the liquid effusion. As in regard to the dilatation of the bronchi as a result of bronchitis, he views the enlargement of the side and protrusion of the intercostal spaces as more than the result of mere mechanical pressure, and the extent to which it takes place distinguishes this affection from simple hydrothorax and pulmonary emphysema, where the dilating causes are simply mechanical, and there is no elevation of the intercostal depressions. We are disposed to agree with Dr. Stokes that in many cases there is a paralysis of the intercostal muscles, if not of the diaphragm, on one side; but we doubt that it is constant, even in chronic cases; and, where it does exist, something should be attributed to the disuse of the muscles from the fixed state of the side, as well as to the paralysing influence of inflammation. We can, however, bear testimony to the truth of the remark, that irritation of the pleura causes spasmodic action of the intercostal muscles, followed by paralysis. In a case of perforation of the lung, with consequent pleurisy, which we had the opportunity of watching closely from its onset, the knotty spastic state of the intercostal muscles was at first most remarkable, and was in a few days followed by relaxation and tympanitic distension; so that the chest on that side became shining and almost without irregularities.

For measuring the chest, with the view to discover dilatation or contraction of the side, and to determine how it changes from time to time, Dr. Stokes has contrived a pair of spring callipers, with an arc graduated in inches and tenths, and with a wooden ball on each extremity. Above the graduated arc there is a spiral spring in a box, by which the balls can be kept applied to the chest during the movements of respiration, which may also be measured on the arc. This instrument has several advantages over the usual method of measurement: it enables one to read off at once the diameter of any part of the chest from front to back, as well as from the spine to the side; and, by fixing one ball on the ensiform cartilage or on the spine of the lowest dorsal vertebra, by moving the other ball to the lower edge of the clavicle or to the upper ridge of the scapula, the depth of the chest may also be accurately compared on the two sides. Still this is an additional piece of apparatus, and, although not a complicated one, it is not likely to be brought into use, except by the more zealous physicians in their hospital practice. Improved methods of gaining knowledge are daily multiplying upon us; but our time is not lengthened in proportion, and we are often constrained to use the simplest method, and that which takes least time, rather than the most complete and accurate.

Dr. Stokes notices a new mode of displacement of the heart to the right side, in consequence of the rapid absorption of a pleuritic effusion on that side. It is supposed that the lungs, being bound down by adhesions, and the chest unable to contract as soon as the fluid was absorbed, the mediastinum, with the heart and left lung, were drawn over to the right side, to fill the vacuity. We doubt that the rapid absorption of the effusion is necessary to produce this result, which may be rather expected when the effusion has remained long, and the lung so bound down in its compressed state that no collapse of the chest can efface the vacuity. We have at present a patient under our care, a boy thirteen years of age, who had a latent pleurisy of the right side, ten months ago: the chest has been contracting during the last four or five months; the respiratory murmur, which is distinct in the upper two-thirds of the right side, does not reach to the lower third, which is quite dull on percussion, and the heart can be seen and felt to pulsate two inches to the right of the sternum.

We will conclude the subject of diseases of the chest with a notice (short, because we have so recently reviewed the subject in connexion with M. Bouillaud's work,) of some of M. Andral's remarks on the auscultatory phenomena of the heart. They are expressed in his peculiar clear style, and evince his usual discretion and liberality of sentiment, although the somewhat exclusive patriotism of his countrymen is, perhaps, also apparent. It is difficult to say whether it is from ignorance of the English language, or from a national partiality to the works of his own countrymen, that M. Andral scarcely notices the researches or opinions of English writers on this subject, although they were published years before those of the French authors to whom he ascribes them. We would hope, for the sake of the fair name of Andral, which stands so high for liberality and candour, that the former reason is the cause of his silence; for he makes an exception in favour of the experiments of the Dublin Committee of the British Association, which had been translated in the Encyclographie. The experiments of Dr. Williams, although nearly the same, and published before those of the Dublin Committee were performed, are not noticed, because, we presume, they had not been translated.

The following are supposed by M. Andral to be the causes of the bellows' and other murmurs heard in the region of the heart:—1. Difficulty in the passage of the blood through the various orifices of the heart. 2. Unnatural reflux of blood through the orifices by which it has already passed. 3. Modification in the play or movements of the valves. 4. Abnormal contraction of the muscular tissue of the heart. 5. Increased force of impulse. 6. The compression of a tumour. 7. Friction between the layers of the pericardium, altered by disease. 8. Chlorotic and anæmic states of the system, acting in some unknown way, producing murmurs in the arteries more frequently than in the heart.

Among the first of these, M. Andral reckons not only morbid constrictions of the orifices, which are undisputed causes in many cases, but also too large a quantity of blood, a change in the size of the cavities of the heart, and a loss of smoothness in its internal surface. The three last appear to us questionable. M. Andral admits that he has seen very few cases in which plethora alone could be regarded as a cause of bellows' murmur. It is not easy to conceive either how enlargement of the cavities alone can produce a murmur. When it occurs with thinning of the walls, it undoubtedly causes a louder and shorter first sound; but this is the reverse of a murmur; and we suspect that, in the cases where a bellows' murmur does accompany this condition, there is either a dilated state of the arterial trunk, or an imperfect closure of an auriculo-ventricular valve. The latter is, according to our experience, a very common cause of abnormal murmurs, and is liable to be overlooked by those who do not duly consider the structure and action of these valves. The lengthening or shortening of any of the tendinous cords, change of structure, or loss of power, or irregular action in any of the columnæ carneæ; rigidity, puckering, or wasting of the laminæ of the valves, may afford a chink through which the blood will be forced, in a sonorous jet, back into the auricle. The cause of sound is intelligible here; but not so with a simply dilated ventricle. We would fully recognize the existence of the second class of causes,—insufficiency of the arterial valves, permitting regurgitation. These were first distinctly described by Dr. Corrigan; but, as we have just remarked, the auriculo-ventricular valves may be the seat of a similar lesion. So also change in the structure of the arterial valves may modify the character of the natural second sound of the heart, which has been fully proved to have its cause in the reaction of the blood on them.

M. Andral has, like some other writers, classed together, in a manner that we cannot think correct, the various murmurs which are obviously produced in the current of blood with the friction sound of pericarditis, the seat of which is external to the heart, and the natural sounds, which are most probably dependent on the solid structure of the heart. Yet these three kinds of sound,—a bellows' or sawing murmur, a friction sound, and the natural double sound,—not unfrequently coexist at the same time; and the manner in which the two former arise and cease, the natural sound remaining the same, or being modified only in its own way, strongly indicate the separate seat of these several sounds, and that the former are rather additions to, than modifications of, the latter. The character of the double sound of the heart may be varied by disease: dilatation renders the first clear and short, hypertrophy long and dull; but such varieties can be always distinguished from complications with murmurs, which, in their smallest degrees, can, by the practised ear, be distinguished to be additions to the natural sounds.

With regard to the eighth class of causes of murmurs, chlorotic or anæmic states of the system, M. Andral expresses a strong bias to the opinion that they produce the sounds in question in consequence of an altered condition of the blood. The remarkable sound heard in the neck, called, from its resemblance to the noise of a toy of that name, "bruit de diable;" and one of an analogous kind, which, from its likeness to the buzzing of a fly, is termed "bruit de mouche," are considered by M. Andral to be of this kind in the carotid arteries; and so convinced is he that they indicate the existence of a chlorotic state of the system, that he is guided by their presence to prescribe steel medicines for the patient. On the subject of the *bruit de diable*, we stated, in a former Number, the opinion of Dr. Ogier Ward, that it is produced in the jugular veins. This opinion has been confirmed by the experiments of the London Committee of the British Association, appointed to investigate the sounds of the heart. The experimenters, Drs. Williams and Todd, state, moreover, in their Report, (*see, in our present Number*, Part IV.) that, so far from being essentially connected with a chlorotic or any other morbid state of the blood, they were able to produce it at pleasure, in the healthiest subject, by a certain degree of pressure with the stethoscope on the jugular veins; and, in this and every other case of its production, it could be stopped or increased at pleasure by various circumstances which would stop or accelerate the current of blood in these veins. It is probable, however, that in chlorotic patients and others, in whom the circulation is rapid, the veins not full, and the blood thin, these sounds may be more readily produced; and that, under such circumstances, the pressure of the strained muscles of the neck, independently of that of the stethoscope, may be sufficient to excite it. Since the Report of the Committee has been published, we have met with several cases in which a continuous buzzing or humming sound was heard in the neck and below the sternal ends of the clavicles; sometimes obviously produced by the pressure of the instrument, but in others without it: in two of these there was an enlarged thyroid gland, in two others aneurism of the aorta pressing on the veins; in all cases, considerable pressure on the jugular veins in the neck stopped the sounds. We think that attention to these sounds may assist in the diagnosis of tumours in the upper part of the chest.

We now turn to the subject of Diseases of the Larynx and Trachea.

On looking through the different works the titles of which are prefixed, we find them all to possess considerable merit, although differing somewhat in their excellencies. The treatise of Mr. Porter is a second and improved edition of a work with which his name has long been favorably associated, and in which many interesting observations and judicious reflections are to be found. Its principal object was to determine, by collected cases and pathological reasoning, what are the affections and circumstances in which the important operation of *bronchotomy* may properly be performed. Ten years of additional experience, improved latterly by the assistance afforded by auscultation, have enabled the author to enlarge and enforce his "Observations," which ought to be carefully studied by every young medical man, who may be called at any moment to the responsibility of using or withholding this momentous resource of surgery. It is much to be regretted that observations valuable as these, should not have the advantage of clearer language and arrangement. There is such a looseness in the style of composition, and such a want of system in the whole work, that many a hasty reader will, we expect, rather derive from it a few vague and unstable ideas than the solid instruction which it ought to afford. There is not even a table of contents.

The treatise of Mr. Ryland is chiefly a compilation, (and a very good one it is,) comprising a great part of the best and most recent information in this and other languages, fairly written, well arranged, and commented on by one who is practically conversant with his subject, and who adds some interesting results of his own experience. This is the most complete treatise of the whole, and we shall take it as the basis of our brief review: it is an enlargement of an essay to which was adjudged the Jacksonian prize of 1835.

The joint work of MM. Trousseau and Belloc is also founded on a history of Laryngeal Phthisis which successfully competed for a prize offered by the Royal Academy of Medicine at Paris. The authors had been engaged in the work before its subject was proposed as a prize-question. They have collected, from various sources, upwards of sixty cases of different affections of the larynx and trachea. They have not failed to extract cases from English writers; but, in their historical notice of those who have contributed to the knowledge of the subject, they too often seem to forget that the English have done anything.

Mr. Ryland's first three chapters contain a brief but clear account of the anatomy of the larynx and trachea, their development and malformations,—the physiology of the larynx and the voice. The fourth chapter is a condensed transcript from Mr. Porter's "Observations" on the general pathology of the mucous membrane. Inflammation changes the natural pale-rose tint to a red, or even purple or deep violet: in the chronic state this redness is lighter, and merely in patches, which are shaded into the natural tint. We have seen, however, in chronic cases occurring in scorbutic subjects, the membrane assuming the deepest red tint. The mucous membrane, and the submucous cellular tissue under it, also become swelled and pulpy in proportion to their laxity: thus the lips of the glottis, when inflamed, often become so much swelled as to impede the passage of air in respiration; while the membranes covering the cricoid cartilage and rings of the trachea are scarcely thickened. The same thing may be observed of œdema and deposition of lymph. Ulceration frequently follows inflammation, and it may attack any part of the membrane: after the acute form, the lips of the glottis and upper parts of the larynx, in the irritation accompanying tubercular consumption, the covering of the cricoid cartilage, vocal cords, and under surface of the epiglottis and posterior part of the trachea, are the most usual seats of ulceration; while aphthous and venereal ulcers more generally occupy the upper parts of the larynx, and extend from the fauces or pharynx. We do not quite understand why Mr. Porter and Mr. Ryland believe these ulcers never to heal by granulations, but only by a contraction of the edges of the sore. The wrinkled stellated appearance of their cicatrices is surely no proof of this; for the same puckering is seen in the scars from ulcerated wounds of the integuments, which never could have healed but by granulation. There seems to be a general tendency in new tissues, when slowly formed, to contract and become condensed for some time after their formation. Mr. Porter describes as the most important form a broad flat ulcer with uneven edges, covered with a bright yellow slough, and disposed to spread rapidly from the arch of the palate or the pharynx down to the larynx. They rarely heal spontaneously; but the patient generally dies from the depraved and cachectic state to which they owe their origin, and which is often connected with a scrofulous or venereal taint. Besides ulcers, MM. Trousseau and Belloc describe erosions of the mucous chorion or epithelium, which may be mistaken for ulcers. If these be examined under water, they will be found to present a villous surface, unusual in the natural state of this mucous membrane. They do not appear to be connected in any way with ulceration; but, as they are only found in cases of pulmonary phthisis, Louis concluded that they are caused by the action of the expectorated matter on the membrane.

The changes of the secretion of the mucous membrane of the windpipe from inflammation are much the same as those of the bronchi, but perhaps more marked. Thus, in the first stage, it is quite thin and acrid, subsequently becomes viscid, and at the acme of inflammation entirely ceases; the secreting crypts and follicles being, we conceive, blocked up by the interstitial effusion. In this particular the mucous membrane contrasts with the serous, and the more so as it is more mucous and complicated with follicular structures. The albuminous exudations and formation of false membranes depend on the character rather than on the intensity of the inflammation: their greater prevalence before than after puberty is well known. Dr. Stokes offers, by way of explanation, the predominance of the white or reproductive tissue at an early age. We see a simpler explanation in the general fact of the greater activity of the nutritive function in the child, the greater freedom with which the plastic material is supplied from the blood, and perhaps also the simpler, finer condition of the mucous membrane at that age; when, not having been long subjected to the various irritations which it has to endure, its follicular apparatus has not yet attained its full activity or development. Hence the effect of inflammation on it will be more like that on serous membranes. In all these cases, however, we must regard the condition of the blood as an element essential to the production of any particular result of inflammation; and this alone, existing in excess, may, although rarely, be sufficient to cause a similar result in after-life.

The inflammatory diseases of the larynx and trachea are severally taken up by Mr. Ryland, in as many chapters. On Acute Laryngitis, Œdema of the Glottis, Erysipelatous Laryngitis, Chronic Laryngitis,

(subdivided into that affecting the soft parts and that affecting the cartilages,) Croup, and Diphtheritis. The other authors place croup in the foremost rank; and certainly its greater frequency and decided inflammatory character would assign to it that place, although the peculiarity of its product marks it apart.

The acute laryngitis of adults frequently commences with symptoms of ordinary sore-throat, accompanied with fever; the pain, constriction, and soreness in the larynx coming on after, with a harshness of voice and frequent husky cough. By pressing the tongue forwards and downwards, the epiglottis may often be seen, erect, thickened, and of a deep red colour; which state of the part often causes a difficulty of swallowing. Then rapidly follow signs of swelling of the membranes of the larynx, and consequent obstruction to the breathing, bringing with it those frightful symptoms of that strangulation in which too often the disease terminates with awful rapidity, sometimes within twenty-four hours of the first attack. Mr. Ryland quotes four cases, illustrative of the character of the disease, and gives a table of twenty-eight, with their results. Of these, ten recovered; but Mr. Ryland justly considers this above the average. The unfavorable cases are not so freely acknowledged by writers as those of recovery. Death probably depends in part on a spasm superadded to the constriction from thickening; for there are generally exacerbations of the dyspnœa which are too sudden to be anything but spasmodic. Sometimes, however, the patient dies in the interval, but not without a duration of the dyspnœa sufficient to produce the lividity, congestions, and effusions of impending asphyxia.

All writers seem to agree with respect to the treatment of acute laryngitis, that it must be of the most prompt and vigorous nature; and, unless it gives very speedy relief, bronchotomy should be performed, to keep the patient alive until the remedies shall have time to act. Free bleeding, general and local, but not to syncope (Dr. Cheyne thinks), mercury and antimony in full doses, may be given a chance for a few hours; but, as soon as ever the dyspnœa becomes distressing, the operation should not be delayed. "As long as bronchotomy is considered an extreme measure, a *dernier ressort*, it will always be performed too late in this disease:" so said Louis. Mr. Lawrence says that it should be done "as soon as the symptoms enable us to determine the nature of the disease." It has generally failed from having been delayed too long; it does, however, sometimes, although rarely, succeed in extreme cases.

"In a case quoted by Dr. Cheyne, the operation was performed by Mr. Goodeve after the pulse had ceased at the wrist, the face suffused, and the lips livid; yet recovery took place. I shall never forget the case of a gentleman, aged upwards of sixty years, who had recently recovered from a violent pneumonia, and was attacked with the most violent form of acute laryngitis, which, baffling all efforts to control it, brought the patient to the jaws of death in little more than twelve hours. It was determined that Mr. Porter should be sent for to operate; but, before he arrived, the patient had become nearly insensible. The operation was proceeded with, but respiration ceased before the trachea was opened. The operator paused,—it was a fearful moment,—and then rapidly opened the trachea; yet no sound of inspiration followed. Applying his mouth to the wound, he inflated the lungs and produced artificial respiration at least seven times, when a loud and rattling inspiration, followed by full and free bleeding, proclaimed the triumph of art." (*Stokes*, p. 234.)

The triumph was but short, however; for we learn from Mr. Porter that, on the night of the second day, bronchitis set in, and the patient died the following morning. When the laryngeal constriction has once begun the process of asphyxiation, the lungs sustain an injury which, with the operation and the remains of the laryngeal disease, constitute a fatal combination against recovery.

All our authors regard œdema of the glottis as inflammatory. Mr. Porter identifies it entirely with acute laryngitis, under the name L. œdematosa. MM. Trousseau and Belloc, who are obviously of the Broussaian school, strenuously maintain that all acute affections of the larynx tending to constriction (we might say that they include chronic also,) are inflammatory, and that spasm is an imaginary cause; for, although the parts be found, as Bayle, Laennec, Cayol, and Dupuytren have observed, perfectly pale after death, the epiglottis, during life, has been seen to be in these cases of a cherry-red colour. But it might be questioned how far this redness (which might be a mere congestion, or the result of the straining and exertion of the parts consequent on spasm,) is always to be regarded as a proof of actual inflammation, where it is followed by no further change. In a case of œdematous laryngitis, given by Mr. Ryland, the epiglottis was not erect, as it is in acute laryngitis; and the absence of pain and fever also implies a distinction from this affection. It approaches to the erysipelatous form of inflammation, acute laryngitis being more phlegmonous. Mr. Ryland gives a good coloured drawing of the larynx of a patient who died from the effects of skinning a glandered horse with a punctured hand. The lips of the glottis are seen almost completely closed by their œdematous distension. In cases of œdema of the glottis that have terminated speedily, the liquid effused has been found to be a limpid serum; in those which have been slower, a semi-purulent matter: so similar differences are observed in the œdema of loose external textures produced by erysipelas. Generally, œdematous laryngitis is excited by chronic disease of the larynx or adjoining parts; but it may be developed independently, as Bayle remarked, especially during convalescence from typhoid fevers; and it may, by producing small abscesses and ulcerations which reach the cartilages, induce laryngeal phthisis. Medicine has even less control over this affection than over the more sthenic form of laryngitis. Local bleeding may relieve the soreness, but it rarely influences the œdema; and, if mercury might do more, there is not time for its action. This is eminently a case for bronchotomy, which should not be delayed when once the disease has declared itself.

Mr. Ryland gives six original cases of laryngitis, which he calls erysipelatous, because erysipelas either preceded or followed it, or was prevailing when the cases occurred. Two coloured delineations are given to shew the inflamed state of the larynx, and effusion of matter and sloughy condition of the submucous cellular tissue: the erysipelas here appears to have been of the phlegmonous kind. Two cases recovered: in one, improvement being accompanied by the appearance of erysipelas on the face; in the other, erysipelas of the face and scalp existed during the whole complaint, and the parts were dressed with mercurial ointment, while calomel was given internally, and leeches applied to the throat. Mr. Ryland suggests that, as the fauces are usually attacked first, the occurrence of pain, heat, and dryness in the throat of an erysipelatous patient, with impeded deglutition, should occasion prompt efforts to stop the progress of the inflammation to the larynx, by leeches behind the angles of the jaw, free blistering of the back of the neck or upper part of the sternum, the inhalation of steam, and internally by mercurial and antimonial medicines, or tonics, according to the state of the general strength and fever. If the voice and breathing, with a sense of constriction in the larynx, announce that this organ is affected, speedy counter-irritation of the external parts, by *acetum lyttæ* or boiling water, must be resorted to. Bronchotomy is spoken of more doubtfully, by both Mr. Porter and Mr. Ryland, in this form of laryngitis; "because the erysipelas, having existed for some days previous to its attacking the larynx, will have considerably lowered the powers of the system, and perhaps have impaired the condition of the brain." There is, however, the case of a patient of St. George's Hospital, narrated in the Medical Gazette for April, 1837, in which this operation was successful in laryngeal constriction suddenly supervening during erysipelas of the face.

Chronic laryngitis is generally a disease very insidious in its origin, often causing little pain but generally a dry hacking cough, and still more constantly some changes of the voice. The inflammation may be in the mucous and submucous tissues only, or it may extend to the cartilages. The latter form, Mr. Ryland thinks, best deserves the name of Phthisis laryngea, from its incurable nature, and the hectic and emaciation which invariably accompany its latter stages. MM. Trousseau and Belloc, however, consider that both forms lead to atrophy, and merit the name of Laryngeal phthisis, which they refuse to admit to be a disease *sui generis*. The treatise of these authors, being expressly devoted to this subject, contains the fullest and most accurate history of chronic laryngitis that has yet appeared, and we wish that our limits would permit us to extract more freely from it. They describe, as the products or complications of this disease, erosions and ulcerations of the mucous membrane, ossification, necrosis and caries of the cartilages, ossification of their peri-chondrium, morbid alterations arising from the preceding lesions, polypi, vegetations, cancerous and tuberculous productions, hydatids, false membranes, and calculi. The description of these lesions occupies fifty pages. We can only notice one or two of the more remarkable. Ossification of the cartilages of the larynx takes place naturally, in advanced life, as in those of the ribs; but chronic laryngitis, of two years' duration, causes the same change in young persons. This is in conformity with a law well developed by M. Andral, that chronic inflammation, or a certain degree of irritation, accelerates those changes in tissues to which time would naturally bring them. The osseous matter is deposited in irregular plates on the surface of the cartilage, and sometimes quite encases it.

Necrosis of the cartilages has scarcely been mentioned by authors. Mr. Porter describes as unusual two cases, and speaks with hesitation regarding the lesion. Mr. Ryland gives a case of necrosis of the cricoid cartilage, and quotes another from Mr. Lawrence. MM. T. and B. state that they have found necrosis in more than half of the fatal cases of laryngeal phthisis which they have examined. The necrosed portion is always denuded, and whenever its death has been caused by disease of some standing, it is ossified also. They consider the ossification to have been caused by ulceration in the vicinity of the cartilage, and when this ulceration reaches the cartilage so degraded by ossification, it soon destroys its little remaining vitality. In support of this view they remark, that in very young subjects, where the cartilages are more *living*, they suffer a less common lesion, caries, rather than necrosis; and the same thing takes place in the arytenoid cartilages and epiglottis, which are never, according to the experience of these authors, ossified. In a case of Mr. Ryland, however, all that was found of the arytenoid cartilages was an ossified remnant of the left cartilage; and Mr. Porter relates another in which an ossified arytenoid cartilage was expectorated. The sequestrum of dead cartilage is not readily thrown off by the living; and as a cause of local and constitutional irritation, or as a foreign body in the larynx, when at length detached, it may be the cause of fatal disorder. Ulceration of the mucous and submucous membrane, and caries, and necrosis of the cartilages, frequently cause considerable engorgement and swelling of the adjacent cellular tissue, which constitutes a common cause of fatal obstruction in the larynx, improperly termed œdema of the glottis. Of nine cases of œdema of the glottis examined after death by these authors, two were acute: one supervened on a violent catarrh, the other after the operation of tracheotomy: seven were chronic, five occurred with necrosis, caries, or ulcers of the larynx, two with ulcerated tumours.

MM. Trousseau and Belloc have found no reason for separating phthisis laryngea from phthisis trachealis, as M. Cayol had done. The causes of laryngeal phthisis are various; a tuberculous or scrofulous constitution more particularly predisposes to it. The excessive use of mercury is considered by Mr. Lawrence, Mr. Porter, and Mr. Wood as a common predisponent cause; typhoid and debilitating diseases generally seem to have the same tendency. The excessive and constant use of ardent spirits may act as both a predisposing and exciting cause. Acute affections of the larynx, excessive exertion of the voice, catarrhs often recurring, repressed eruptions, blows, falls, wounds, chills, extraneous bodies introduced, may act as exciting causes. Four species of the disease are enumerated, simple, syphilitic, cancerous, and tuberculous. These are illustrated by numerous examples.

Of the symptoms, the earliest and most constant is a change of the voice. The degrees and kinds of hoarseness are very various. The stridulous variety generally implies a worse state than a mere loose or mucous hoarseness. In a great many patients the voice is very unequal, but patients by habit instinctively moderate these inequalities except on any unusual exertion of the voice when its cracked tones are very manifest. The hoarseness is liable to increase from various circumstances, particularly weakening causes and vocal exertions, and may sooner or later terminate in aphonia. When the loss of voice is gradual, the prognosis is most unfavorable; for this is generally the result of a progressive destruction of the vocal apparatus. The cough is short, frequent, and generally dry in the early stages. Later there is sometimes a very peculiar kind of loose *belching* cough, which is supposed to depend on imperfect closure of the glottis. In more than half the cases of ordinary laryngeal phthisis, there was no pain throughout the duration of the disease. Painful deglutition is often complained of; which MM. T. and B. ascribe to the circumstance that the aryteno-epiglottic ligaments, and the arytenoid cartilages, which form the framework of the front of the pharynx, are covered by membranes which are very commonly the seat of inflammatory engorgement. At the same time the larynx may suffer outward pressure without pain, because the same parts are protected by the hyoid bone and thyroid cartilage. There is little within reach of inspection, but the epiglottis, and even this cannot often be seen: our authors have twice succeeded on making the patient utter cries during the examination. A speculum for the larynx is much needed. The crepitation felt on pressing the larynx, said by M. Laignelet and others to be a sign of laryngeal phthisis, MM. T. and B. find may be generally produced in the healthy larynx. Feeling the glottis, which has been recommended by some authors, is a thing impossible. To feel even the epiglottis is a matter of considerable difficulty; so violent are the convulsive efforts excited by the touch: so that except very palpable changes, little is to be learnt by feeling. The signs of respiration are next in value to those of the voice. As the disease advances, the patients are attacked in the night with paroxysms of dyspnœa, which leave them in the intervals only with their breathing short on exertion. These paroxysms become more severe, amounting to orthopnœa, and then the respiration in the interval is noisy. The disease advances; the frightfully aggravated paroxysms threaten imminent suffocation; and then the orthopnœa does not subside in the intervals. Death generally ensues in fifteen or twenty days after the commencement of the orthopnœa. Bayle and Thuillier have described the inspiration being much more difficult than the expiration, as an essential character of an œdematous state of the ligaments of the glottis. This MM. T. and B. have not found to be the fact. The gradual attacks of laryngeal paroxysms of dyspnœa and the manner in which the voice is affected by them, will serve to distinguish them from pulmonary asthma. MM. Trousseau and Belloc do not appear to have attempted to find any signs of laryngitis by auscultation. Dr. Stokes describes, as the stethoscopic signs of chronic laryngitis, a harshness in the laryngeal sounds of respiration, giving the idea of a rough surface; this is perceptible even when the breathing is not stridulous. In some cases, above the alæ of the thyroid cartilage there may be heard a rhonchus, like the sound of a small valve in action combined with a deep humming sound. It is sometimes perceptible only on one side; and always disappears as we descend to the bronchial tubes. When the obstruction is considerable and the respiration prolonged, a remarkable feature, mentioned by Dr. Stokes, is the feebleness of the vesicular murmur as compared with the violence of the inspiratory efforts. The feebleness is observed throughout the chest, and not, as in the case of the pressure of a tumour, on one side chiefly.

MM. Trousseau and Belloc fairly establish that laryngeal phthisis does occasionally arise, run a long course, and terminate fatally, without being complicated with any other organic lesion. They admit that these cases are rare, and that generally chronic laryngeal disease is secondary, and most commonly an addition to pulmonary phthisis. But they bring several well marked cases to prove that, not very rarely, the laryngeal affection is primitive, and pulmonary or other organic tubercular affection is ingrafted on it; these cases they think may also properly be called laryngeal phthisis; although they view them as the result of a tuberculous diathesis, now manifesting itself in one set of organs, now in another; now long affecting the mucous membrane and Peyer's glands in the intestines, or the membranes of the larynx; now developing itself in the lungs and more rapidly destroying life. There is sometimes great difficulty in cases of laryngeal disease, in determining whether pulmonary disease also exist or not. The noisy laryngeal respiration, the absence of voice, the occasional presence of bronchitis, obscure the physical signs by which lesions are to be detected in the lungs; and these lesions may be of that kind which, even without these disguising circumstances, can be discovered only by the most experienced auscultators. When, however, the pulmonary disease is more advanced, the copious purulent expectoration, emaciation, and night sweats, which rarely attend simple laryngeal lesions, with dulness on percussion in some part of the chest, generally make the case pretty clear.

Simple laryngeal phthisis is in its milder forms by no means incurable. MM. Trousseau and Belloc give instances in which a temporary cure was effected even where subsequent tubercular disease in the lungs proved the existence of a scrofulous taint. Rest to the organ of the voice is quite indispensable, and these authors have found that the rest ensured by speaking only in a low whisper is sufficient. This is a far more practicable rule than that of absolute silence and it is quite reasonable, for the larynx is no more concerned in whispering than it is in breathing: it is the voice that constitutes its labour. And here we would remark that the large and extended vibrations of the male voice may constitute one cause of the much greater frequency of laryngeal disease in men than in women. Of depletive measures suited to the early stages of chronic laryngitis, MM. T. and B. give a preference to venesection; but other modes of bloodletting may be better suited to particular cases. Blisters, setons, and especially counter-irritation by tartar-emetic or caustic potass, in the neighbourhood of the larynx, or at the back of the neck, are of more permanent efficacy than bleeding; and are especially suited to the disease when established. When pain or cough is troublesome, it is proper to quiet them by narcotic applications; and frictions of extracts of stramonium and belladonna are recommended by our authors.

The most effective remedies, however, are those applied to the diseased part itself. A solution of nitrate of silver, in the proportion of from one to two parts dissolved in four parts of distilled water is the application which MM. T. and B. have employed with the greatest success. The solution may be applied to the epiglottis and upper part of the larynx by a small roll of paper bent towards its end and well moistened with the solution. A more effectual mode is with a small round piece of sponge firmly attached to a long piece of whalebone, bent at an inch from the sponge to an angle of 80°. The patient's mouth being opened wide, and the tongue pressed down with a spoon, the sponge is passed to the top of the pharynx; as soon as it reaches the fauces, a movement of deglutition takes place, which carries the larynx upwards, at which moment the sponge is brought forward and squeezed under the epiglottis, and the solution freely enters the larynx. Convulsions of cough, and sometimes vomiting ensue, but the application causes no pain. A less disagreeable mode of applying the solution, is by a small silver syringe with a tube five inches long bent at the end; this is filled to a fourth of its capacity with the solution, and the remaining three-fourths with air. The extremity of the tube is carried beyond the epiglottis and the syringe, being discharged forcibly, throws the solution into the larynx in a finely divided rain, in consequence of the presence of air in the syringe. The patient is then made to swallow and rinse his mouth with a solution of salt or a muriatic acid lemonade to decompose the remains of the nitrate; and this precaution should be adopted in those cases also where this remedy is applied in substance. In six cases of aphonia with various degrees of "chronic laryngitis" this treatment proved successful; in the slighter cases, in the course of a few days: in more inveterate cases, where the disease had lasted for three or four years, the cauterization (as they term this application) had to be applied repeatedly during a month or more. It produced considerable improvement in three other cases, two of which proved afterwards to be tuberculous. It may be a matter of some doubt whether the aphonia in some of these cases at least be not the result of relaxation rather than inflammation, and the irregular menstruation of some of the patients also countenances the idea that it may be in some measure hysterical. However, this treatment succeeded where all kinds of other means had failed; and we are indebted to these authors for making it known and for the assurance that it may be safely employed in so ticklish a neighbourhood.

Another method of applying remedies to the diseased parts is by the insufflation of powders, as recommended by Aretæus for angina maligna, and by M. Bretonneau for diphtheritis. The method recommended by MM. Trousseau and Belloc is simple. The powder is put into one end of a reed or glass tube, two lines in diameter and eight or ten inches long, the other end being carried as far back as possible into the mouth, after a complete expiration the patient closes his mouth and makes a sudden forcible inspiration, in which the air carries some of the powder into the larynx and trachea. It excites cough which should be restrained as much as possible to prevent the too speedy expulsion of the medicament. This insufflation is to be repeated several times during the day according to the nature of the case. The powders employed in the various cases are—sugar; sub-nitrate of bismuth (which may be used pure;) calomel with twelve times its weight of sugar; red precipitate and the sulphates of zinc and copper with thirty-six times their weight of sugar; alum with twice its weight of sugar; acetate of lead with seven times, and nitrate of silver with twenty-four, thirty-six, or seventy-two times its weight of sugar. The powders ought to be made quite impalpable. The least roughness or perceptible fragment of a crystal excites such efforts of cough as ensure the expulsion of the powder. Of the powders the sub-nitrate of bismuth is the mildest; and may be used with safety and often with advantage in every form of chronic laryngitis, even in that accompanying pulmonary phthisis. Nitrate of silver is the most effectual in erythematous laryngitis, with erosions or even ulcerations. Calomel and red precipitate have proved beneficial in ulcerations whether syphilitic or not, but they may not be repeated at first more than twice or thrice in a week.

MM. Trousseau and Belloc have found repeated mild mercurial courses effectual in several cases of chronic laryngitis, not only those of syphilitic origin, but more decidedly in that kind. In this respect they follow in the track of British practitioners. In fact, their methods of treatment are far more active and like the practice of this country, than is usual in

France; and we should not be surprised to find that our volatile neighbours will soon outdo us in the boldness of their treatment. Laennec, with his large doses of tartar-emetic, and Bouillaud, with his bleedings "coup sur coup," have already led the way.

We return to our British authors. Mr. Porter and Mr. Ryland give good descriptions of the symptoms and history of croup; but as these do not present any new matter, we must pass them over. It is judiciously observed by Dr. Stokes that much confusion has arisen in consequence of authors not having distinguished between the two forms of croup, which he designates *primary* and *secondary*, which are diseases of a very different type and requiring quite distinct modes of treatment, yet, in consequence of having the same name, the most perplexing contradictions have arisen. In *primary*, or true inflammatory croup, the air passages are primarily affected and there is present an inflammatory symptomatic fever, requiring and sometimes cured by antiphlogistic means. In *secondary* croup, the *diphtherite* of Bretonneau, the laryngeal affection succeeds to disease of the pharynx or mouth; if fever be present it is generally of a typhoid character, requiring tonics, revulsives, and stimulants, instead of antiphlogistics. Primary croup is sporadic or endemic, but never contagious; it chiefly affects children. Secondary croup is always epidemic and (Dr. Stokes thinks) contagious, and often attacks adults. In primary croup the exudation of lymph spreads from below upwards, the pharynx being healthy, and deglutition generally easy. In secondary croup, the exudation spreads from the glottis downwards, the pharynx being affected, and severe dysphagia often present. In primary croup, catarrhal symptoms often precede the attack; pulmonary inflammation is commonly combined with it; and the breath is seldom fætid. In secondary croup, the laryngeal symptoms supervene without preceding catarrh, the lungs are seldom affected, and the breath has a characteristic fætor. The only pathological resemblance between these two affections, inflammatory croup and diphtheritis, is the secretion of plastic lymph or false membrane from the laryngeal mucous membrane.

Dr. Stokes thinks, in oppposition to the opinion of Dr. Meriadec Laennec, that stethoscopic examination may be useful in true croup. In the early and more tractable stage of the disease, the breathing is not too stridulous to prevent us from hearing in the lung loud sonorous and mucous rhonchi diffused through the different orders of bronchial tubes, sometimes with peripneumonic crepitation with some dulness on percussion. The act of vomiting being often followed by a temporary suspension of the stridulous breathing, supplies an opportunity of hearing these signs more distinctly.

On the subject of treatment of croup our authors differ considerably. All recommend bleeding and antimonial emetics at the onset. Mr. Porter prefers venesection, especially jugular; and considers leeches highly objectionable. Mr. Ryland recommends leeches in the majority of cases, "because the disease more frequently occurs in weakly children." Dr. Stokes advocates general and local bleeding "as assistants to the principal remedy, which is the tartar emetic;" but objects to opening the jugular vein, lest the vomiting should afterwards burst the incision, and cause dangerous hemorrhage. Messrs. Ryland and Porter recommend tartarized antimony in an emetic dose only at first, the latter trusting chiefly to nauseating doses afterwards, and the former exclusively to calomel. Dr. Stokes follows Dr. Cheyne in recommending doses to be given sufficient to produce vomiting at least once in every three quarters of an hour, and continued for several hours according to circumstances: the mercurial treatment he believes "to be insufficient and unnecessary." If we may be permitted to give a casting vote we would state our experience to be generally against bleeding further than by leeches, and this only at the first attack; and, although we trust more to tartar emetic in full doses (about a quarter of a grain every half hour) than to any other remedy, yet we have seen such depression rapidly produced by it, that we make it a rule to give calomel also, to the more durable action of which we entrust the case, when we are constrained to give up the tartar emetic. All agree, however, as to the inexpediency of attempting bronchotomy in croup. In the early stages of the disease there is too good a chance of the medical remedies being successful to warrant the interference of surgery; and by the time their efficacy begins to be doubtful, the condition of the bronchi and lungs, as well as the state of the system generally, is commonly beyond the reach of relief from tracheotomy. The few instances on record in which it has succeeded, appear to be quite exceptional cases. The same remark applies to the epidemic adult croup, or diphtheritis. The remedies most to be relied on in this affection, which appears to be a specific form of anginose inflammation, are mercury, and topical applications. "The topical applications on which reliance is chiefly to be placed are muriatic acid, nitrate of silver, and alum; and their mode of action seems to be the exciting of such a degree of common inflammation as shall supersede and set aside the special inflammation under which the patient is labouring. The safest of these, and the one which has effected the greatest number of cures, is alum: it may be reduced to a paste by admixture with water or honey, and it should then be applied to the inflamed surfaces by means of a small brush or the handle of a tea-spoon. Its effect is to detach the false membranes, and to repress the exuberance of the swelled and inflamed mucous surface. One application is seldom sufficient; it should be applied frequently till the albuminous exudations cease to present themselves, and till the parts are reduced to their natural colour and volume." (*Ryland*, p. 174.)

Mr. Ryland has an interesting chapter on "Spasm of the Glottis," which comprehends two different kinds of nervous affection, the *laryngismus stridulus* of Mason Good, referred by Dr. Hugh Ley to a paralysis of the inferior laryngeal nerves, generally from the pressure of glandular and other tumours, and spasm of the glottis from pressure on the larynx and trachea, or on their nerves. The last addition "*on their nerves*," destroys the pathological distinction between these two classes of cases; and it appears that Mr. R. arbitrarily applies the first exclusively to the crowing or croup-like inspiration of children; and the latter to similar affections in the adult as well as to obstructions of breathing from the pressure of tumours on the windpipe. Having recently noticed this subject we shall only observe that Mr. Ryland defends the views of Dr. Ley against the objections of Dr. Marshall Hall; and considers them to be confirmed and illustrated by the observations of Drs. Montgomery, Kopp, and Kirsch, on the affection of infants called by the latter authors thymic asthma.

Another class of nervous affections of the larynx are presented by those persons, generally females, who suddenly, on some strong mental excitement or bodily impression, lose their voice, and sometimes as suddenly recover it. In other similar subjects, a violent convulsive cough comes on in paroxysms, sometimes accompanied with stridulous breathing and severe dyspnœa. Irregularities of menstruation, or the occasional presence of globus, hysteric convulsions, or some other indication of the protean malady, stamp some of these cases as belonging to the undefinable disease *hysteria.* But in other instances, there is no trace of any affection of the sexual function, or of the peculiar nervous symptoms that are commonly associated with it: but the symptom is a solitary nervous one. We know a lady not in the least hysterical, who for several years past has been subject to a loss not only of her voice, but even of the power of articulation, on any sudden impression on the body or mind, and the aphonia will continue for a period varying from a few hours to several days, and then cease as abruptly as it came on. For a long time an electric spark was efficient in restoring to this lady the use of her voice; now that fails; and swallowing a morsel of ice generally succeeds. The loss of voice is accompanied with a feeling of constriction in the chest and some oppression of the breathing, which is very indistinctly heard on auscultation. Hence the affection would appear to be spasmodic. Dr. Stokes judiciously remarks that spasmodic affections of the larynx most commonly occur in connexion with organic disease of the larynx or lung; but in a few cases, an affection at first spasmodic passes into one with structural change. He gives one case in which the patient, after many months of suffering from various hysteric and nervous symptoms ending with a convulsive cough which was temporarily relieved by antispasmodics, died soon after with symptoms of suffocation; the only disease found was an abscess of the size of a Spanish nut involving the cricoid cartilage. In two other cases death ensued in consequence of affections of the brain supervening on long continued convulsive cough. In a third case of frightful paroxysms of cough, acute hydrocephalus came on and was arrested by antiphlogistic treatment and mercury.

We have not space for any notice of the subjects of tumours and wounds in the larynx and trachea, which are fully discussed in the works of Mr. Porter and Mr. Ryland. The latter gives two plates shewing polypi of the larynx, taken from preparations in the museum of King's College, London.

The laryngitis *from swallowing boiling water* or *corrosive liquids*, and, as Mr. Ryland adds, *from the inhalation of flame*, is generally complicated with considerable injury to the mouth, fauces, and occasionally to other parts of the body; and the success of treatment, medical and surgical, will much depend on the freedom of the case from these complications. The access of the irritant to the larynx itself in these cases generally arises from the convulsive actions caused by the unexpected pain suddenly excited. A child, unconsciously going to suck water from the mouth of a boiling tea kettle, a person taking sulphuric acid in mistake, can scarcely actually swallow such bodies, but, in their violent efforts to reject it, enough passes under the epiglottis to produce the inflaming effects. Yet, Mr. Porter remarks, strong sulphuric acid is sometimes quietly swallowed by the determined suicide, without injuring the larynx at all, and, strange to say, without causing much pain. This we would explain by its property of coagulating the animal matter of the mucus with which the passages are covered, and which thus affords a temporary shield against its corrosive effects. Mr. Ryland remarks, that one reason why tracheotomy has not been followed by success in many of the class of cases which we are considering is, that it has not been followed up by the means proper to subdue laryngeal and bronchial inflammation, which go on after the operation just as much as before it. The object of the operation is not to cure the disease but to supply air until the inflammation shall have been subdued by mercury and other remedies. In most of the recorded cases of recovery this treatment was persevered in after the operation. Mr. Ryland relates three cases which render it very probable that the inflammation affecting the air passages in some instances of burns, particularly from the upper clothes taking fire, is caused by the inhalation of the flame, or at least of very hot air. In these examples the face and neck were much burnt; and the inflammation in the air passages was most intense at their upper parts. We think, however, that the severe bronchitis, attending extensive burns and scalds also, is more commonly dependent on sympathetic or constitutional irritation; as we see the same inflammation accompany exanthematous and other severe fibrile disturbances, independently of any direct irritation of the bronchial membrane.

On the manner and result of foreign bodies getting into the air-passages, we quote the following general statement of Mr. Ryland, which is at once clear and concise.

"Preparatory to speaking or laughing, a full deep inspiration is necessary, and the air is very commonly drawn through the mouth; the edges of the glottis are at this moment widely separated from each other; the mind, interested in conversation, is off its guard; and therefore a particle of food, a plum-stone, or whatever the mouth contains, is easily taken with the current of air into the larynx or trachea. If the intruding body be of small size and light, a severe fit of coughing will, in all probability, soon drive it back again through the rima with considerable force; but, if it is heavy, broad, oblong, or of an irregular figure, it may become permanently fixed in the aperture or in the ventricles of the larynx; it may remain in the trachea; or it may fall into one of the bronchi. The external qualities of the extraneous body will therefore regulate, in a considerable degree, the situation which it will assume and maintain in the air-passages; and its situation will, in like manner, influence the nature of the symptoms that will result from its presence. The consequences of this accident may be, 1st, instant death, if the foreign body become impacted within the rima glottidis, or in the larynx immediately below this point;—2d, pain somewhere in the course of the trachea, severe dyspnœa, and frequent convulsive cough, followed by death, generally in three or four days after the accident, if the foreign body be small and light, and move with the air in respiration backwards and forwards along the trachea; and, 3d, symptoms resembling those of asthma and suffocative catarrh, which may terminate eventually in death, or in the expulsion of the irritating substance during a fit of coughing: in such cases the body is commonly heavy, and has fixed itself in one of the bronchi or at the entrance of some of their ramifications." (P. 279.)

To detect the presence of a foreign body in the air-passages, auscultation may often afford a most valuable assistance. Its application to this class of accidents is chiefly due to Dr. Stokes, and several lives have been already saved by tracheotomy, performed under the guidance of the signs discovered by auscultation. The diagnosis is to be made rather by a comparison of the history of the case and of the collective and successive signs than from any one phenomenon in particular; and in this way we may generally arrive at a distinction between the effects of the presence of a foreign body in the air-passages and those of any disease that may resemble them. If a body be impacted firmly in the larynx, as in one of the ventricles, it will produce more or less violent and incessant attacks of cough and dyspnœa, in which auscultation finds the lungs to be sound and the larynx to be the seat of the constriction; the permanency of which, together with the history, will point out the nature of the case. When a foreign body is in the trachea, it generally gets into one of the chief bronchial tubes, (almost always the right, because this is larger, and lies more in the axis of the trachea;) and here will produce signs of obstruction and local irritation, which may be quite distinctive. When the body is smooth and roundish, as a plum-stone or bean, it may from time to time entirely block up the bronchus, and the consequent sudden and total absence of respiration on that side only, whilst the sound on percussion remains good, will pretty plainly shew the nature of the affection. The evidence becomes stronger when, on violent coughing, the air is suddenly heard again to enter the lung, and when the increase of laryngeal irritation implies that the body has been moved back into the trachea. The alternation of one set of these signs with the other, Dr. Stokes remarks, is not met with in any other case, and cannot fail to point out the source of the evil. When the foreign body is of an irregular shape, as a tooth or piece of bone, it will not completely block up the bronchus; but the constant irritation and secretion, with its accompanying rhonchus, which it produces there alternately with violent laryngeal cough when it gets upwards, together with the sudden occurrence of the symptoms with other features in the history of the case, will generally denote its nature. When the presence of the foreign body has been clearly made out, and sufficient trial given whether the natural forces can expel it, it should be removed by an operation without further delay; as its remaining in the ventricles, even although it do not obstruct the breathing, will not fail, sooner or later, to cause serious structural mischief, both in them and in the adjoining lung.

We have room only for a few more observations on the subject of bronchotomy, which we found to be indicated in several of the conditions of the air-passages which we have reviewed, and which experience has in many instances proved to be successful. Bronchotomy is the generic term, including laryngotomy, in which the opening is made in the membrane between the thyroid and cricoid cartilages; and tracheotomy, in which the trachea, with one or more of its rings, are cut. The former is the easier and safer operation, because there is little to cut through, and scarcely any risk of hemorrhage into the tube: it is, however, not effectual when the larynx is diseased, and the difficulty of keeping the aperture free, and of keeping a tube in it, on account of the extreme irritability of this part, render it ineligible, except in the more temporary cases of œdema or spasm of the glottis, and the like. Tracheotomy is not only more difficult to perform, on account of the depth of flesh to be cut through,—amounting, in cases of fat, œdema, or other swelling, to one or two inches,—but also more hazardous, from the risk of hemorrhage, which, taking place in the trachea, has in several instances caused instantaneous suffocation. The precautions necessary to prevent this and other untoward issues, and directions for the management of the operation, are fully given in the works of Messrs. Porter and

Ryland, to which we must refer our readers, especially young surgeons, who ought to be always prepared against an emergency in which any delay may be fatal, and where there will be no time to consult authorities. Mr. Porter is the more original writer, and speaks as one who has experienced the things which he describes, although not in the clearest language. Mr. Ryland has borrowed freely from him and from others, but he succeeds in placing his subjects distinctly and concisely before his readers; and, as we have before remarked, his work, although chiefly a compilation, is the most complete treatise that we have seen on the subject. There are a few physiological remarks in it which need revision: for instance, in the section to which we have just been adverting, he speaks of the left ventricle of the heart being paralyzed by the circulation of imperfectly arterialized blood in the brain; a notion, with regard to asphyxia, quite inconsistent with the best established views of the present day.

---

